# Gut microbiome-targeted therapies in liver cirrhosis: a protocol for systematic review and meta-analysis

**DOI:** 10.1186/s13643-022-02059-3

**Published:** 2022-08-30

**Authors:** Honglin Jiang, Yan Peng, Wei Zhang, Yue Chen, Qingwu Jiang, Yibiao Zhou

**Affiliations:** 1 Fudan University School of Public Health, Building 8, 130 Dong’an Road, Shanghai, 200032 China; 2 Key Laboratory of Public Health Safety, Fudan University, Ministry of Education, Building 8, 130 Dong’an Road, Shanghai, 200032 China; 3 Fudan University Center for Tropical Disease Research, Building 8, 130 Dong’an Road, Shanghai, 200032 China; 4 Department of Nutrition, Beijing Fangshan District Liangxiang Hospital, No. 45 Gongchen Street, Fangshan, Beijing, 102401 China; 5 Department of Reference, Medical Library of Fudan University, Building 8, 130 Dong’an Road, Shanghai, 200032 China; 6 School of Epidemiology and Public Health, Faculty of Medicine, University of Ottawa, 600 Peter Morand Crescent, Ottawa, Ontario K1G 5Z3 Canada

**Keywords:** Liver cirrhosis, Probiotics, Prebiotics, Synbiotics, Fecal microbiota transplantation

## Abstract

**Background:**

Microbiome-targeted therapies (MTTs), including probiotics, prebiotics, synbiotics, and fecal microbiota transplantation (FMT), have been proposed as a potential treatment for cirrhosis via modulation of gut microbiome, while the impact of gut microflora alteration on liver function in cirrhosis trajectory is unclear, and no related systematic review has been published. We aim to comprehensively assess the effects of MTTs in patients with liver cirrhosis.

**Methods:**

We will search databases of MEDLINE, EMBASE, and Cochrane Central Register of Controlled Trials (CENTRAL) with no time restriction. Only randomized controlled trials published in English will be included. Two independent reviewers will be responsible for study identification and selection, data extraction, and risk of bias assessment, with discrepancies resolved by consensus or referral to a third author. Heterogeneity of studies will be examined using Cochrane Q-test and *I*^*2*^ statistics. The data will be pooled using either a fixed- or random-effects model based on *I*^*2*^ statistics. The results will be presented as risk ratios (RR) or mean differences (MD) with 95% confidence intervals (CI). We will perform subgroup analysis on the type of MTTs and assess the reporting biases. Sensitivity analysis will be conducted to test the stability of each outcome result.

**Discussion:**

There is no current study about the role of MTTs in developing the liver function, and the therapeutic effects of MTTs are inconsistent. By investigating the liver-specific indicators when treating with multiple MTTs on course of cirrhosis, our findings will give more conclusive and stronger evidence about the efficacy of MTTs and provide new insight into the action mechanisms of these MTTs.

**Systematic review registration:**

PROSPERO CRD42021253198.

**Supplementary Information:**

The online version contains supplementary material available at 10.1186/s13643-022-02059-3.

## Background

Liver cirrhosis is traditionally regarded as the irreversible end-stage of liver disease caused by long-term damage of liver [1]. It is characterized by liver fibrosis and portal hypertension, and can lead to serious, life-threatening complications such as gastroenterological bleeding, hepatic encephalopathy (HE), or liver failure [2]. The prevailing aetiologies of cirrhosis include hepatitis B, hepatitis C, alcoholic liver disease, non-alcoholic fatty liver disease (NAFLD), and schistosomiasis [3]. Although recent data suggest that cirrhosis regression or even reversal is possible [1], there is still no clear consensus on cirrhotic treatment. Liver transplantation may be the only curative option for patients with severely decompensated cirrhosis.

Recently, emerging evidences have indicated that perturbation to the gut microbiome is linked to pathogenesis and prognosis of numerous chronic liver diseases [4–9]. Fecal dysbiosis resulting from the liver injuries can in turn deteriorate liver function by provoking systemic inflammation and metabolic abnormality, thus facilitating the development of liver cirrhosis and its various complications. Cirrhotic patients are observed to have changes in the composition and function of gut bacteria, suggesting microbiota as a novel biomarker of cirrhosis [10]. Based on this, there is an increasing interest in human gut microbiome to serve as a potential therapeutic target for cirrhosis intervention.

Microbiome-targeted therapies (MTTs), namely probiotics, prebiotics, synbiotics, antibiotics, and fecal microbiota transplantation (FMT), have been proposed as a therapeutic option for cirrhosis by the manipulation of the gut microbiome. Probiotics are defined as live microorganisms of human origin that exert a health benefit on the host when consumed adequately [11]. Both prebiotics and synbiotics were introduced by Gibson and Roberfroid in 1995 as an alternative to probiotics [12]. Prebiotics are indigestible food ingredients that improve a host’s health condition by selectively stimulating the growth or activity of microorganisms. Synbiotics are a synergistic combination of probiotics and prebiotics. Currently, FMT becomes a promising option for gut microbiota editing and shows a superior impact on alteration of the intestinal barrier function than multi-biotics based supplements [13]. It transfers a fecal microbiome from a healthy donor into the intestinal tract of a patient to promote the proliferation of beneficial microbiota and ameliorate dysbiosis.

Many studies have reported the efficacy of MTTs on the outcomes of several cirrhotic complications (e.g., HE and variceal bleeding), supporting the role of gut microbiota in cirrhosis progression. However, the results of previous studies are inconsistent. Over the past decade, a few meta-analyses that examined the independent effect of pro-/pre-/synbiotics or the combining effects of different types of MTT on cirrhotic patients with minimal hepatic encephalopathy (MHE) or overt HE have shown conflicting conclusions [14–18]. These reviews are limited to a single type of MTT (e.g., only probiotics or prebiotics) or focusing on one complication of cirrhosis (mainly HE or MHE) other than cirrhosis itself. As a novel therapeutic strategy for cirrhosis, FMT has not been systematically evaluated in previous studies. In addition, there is no quantitative review assessing the effects of MTTs on liver function and the severity in cirrhosis.

Therefore, we plan to conduct a systematic review and meta-analysis to provide the most current evidence for the effects of MTTs (probiotics, prebiotics, synbiotics, and FMT) compared to placebo, usual treatment, or no treatment on key liver-specific outcomes in patients with liver cirrhosis. Antibiotics is not considered in the study for their mechanism of action and negative impact on the intestinal microbiota [2] that are much different from other MTTs. This study will emphasize the role of MTTs acting in the improvement of liver function and the severity of cirrhosis and may provide new insight into the action mechanisms of these MTTs.

## Methods/design

### Study design and registration

The systematic review and meta-analysis is registered on PROSPERO (https://www.crd.york.ac.uk/prospero/, CRD42021253198). We will perform the study in full accordance with the Preferred Reporting Items for Systematic Reviews and Meta-Analyses Protocols (PRISMA-P) [19], shown in an additional table (see Additional file 1).

### Criteria for considering studies for this review

#### Types of studies

We will include randomized controlled trials (RCT) only, regardless of their blinding, study design (parallel or cross-over), and publication date, in our primary analyses. For cross-over studies, data from the first phase will be used for analysis. Multi-arm trials that contain eligible intervention and control groups will be included. Only studies published in English will be considered, which is recognized as a limitation. Full journal publication and peer review is required. Gray articles including conference papers and unpublished studies will be considered for inclusion if providing adequate information on the methods and results. Observational studies, cased reports, study protocols, letters, editorials, comments, and animal studies will be excluded from this study.

#### Types of participants

Patients diagnosed with liver cirrhosis using any recognized diagnostic criteria will be included, regardless of sex, age, etiology, severity of disease, and complications at baseline.

Patients with comorbidities at baseline, which are independent of cirrhosis that affect intestinal homeostasis (e.g., metabolic disorders or gastrointestinal complications induced by other hepatic diseases) will not be considered. We also exclude liver transplant recipients, or participants who were receiving antibiotics for treatment of spontaneous bacterial peritonitis (SBP), pre-treatment of FMT, or any other purposes.

#### Types of interventions

1. Probiotics, prebiotics, or synbiotics at any dose, formulation, frequency, duration, and route of administration, given in combination or separately.
2. FMT, defined as the administration of fecal material containing distal gut microbiota from a healthy donor to the gastrointestinal tract of a cirrhotic patient.

#### Types of comparators

Placebo, usual treatment (except antibiotics, probiotics, prebiotics, synbiotics, and FMT), or no intervention.

Studies that are without a control group or use any other gut microbiome-targeted therapies as comparison, including standard of care (SOC), i.e., lactulose (prebiotics) and add-on rifaximin (antibiotics), will be excluded.

#### Types of outcomes

We will assess the outcomes at the maximum duration of follow-up. Studies that lacked baseline data to measure the outcome changes will be excluded. If the pre-specified outcomes are not measured at the final visit, the last available data will be extracted.

Primary outcomes

1. Development of cirrhosis: incidence of HE, model for end-stage liver disease (MELD) score, Child-Turcotte-Pugh (CTP) score
2. Changes in liver function: alanine aminotransferase (ALT), aspartate aminotransferase (AST), albumin (ALB), and bilirubin (BILI)

Secondary outcomes

1. Changes in cytokine level: tumor necrosis factor (TNF)-α, interleukin (IL)-1β, IL-6, IL-8, and IL-10
2. Changes in other biochemical outcomes: endotoxin, ammonia, and white blood cell counts (WBC)
3. Serious adverse events (SAE): the serious adverse events will be defined as cause-specific death, life-threatening medical occurrence, or withdrawals due to adverse events. The number of participants who developed any serious adverse events will be retrieved

### Search strategies for identification of studies

We will search the following electronic databases with no time restriction: MEDLINE Ovid, EMBASE Ovid, and Cochrane Central Register of Controlled Trials (CENTRAL) in the Cochrane Library. We will use controlled vocabulary (such as MeSH term), keywords, and their synonyms as search terms. The search strategy for MEDLINE in Ovid is shown in Table 1. The syntax will be adjusted for the other two electronic databases.

**Table 1:** Example of search strategy for MEDLINE in Ovid

No.	Search term
1	exp Liver Cirrhosis/
2	((hepatic or liver) and (fibrosis or cirrhosis or cirrhotic)).ti,ab.
3	1 or 2
4	randomized controlled trial.pt.
5	controlled clinical trial.pt.
6	random*.mp.
7	placebo.ab.
8	trial.ab.
9	groups.ab.
10	drug therapy.fs.
11	4 or 5 or 6 or 7 or 8 or 9 or 10
12	(humans not animals).sh.
13	11 and 12
14	exp Probiotics/
15	exp Lactobacillus/
16	exp Bifidobacterium/
17	exp Lactococcus/
18	exp Bacillus/
19	exp Enterococcus/
20	exp Saccharomyces/
21	(probiotic* or lactobacill* or lactococc* or bacillus or (enterococcus faec*) or saccharomyc* or VSL* or yog?urt or (bifidus or bifidobacter*)).mp.
22	14 or 15 or 16 or 17 or 18 or 19 or 20 or 21
23	exp Prebiotics/
24	exp Oligosaccharides/
25	exp Inulin/
26	exp Lactulose/
27	exp Fructose/
28	(prebiotic* or fructan* or fructo* or oligofructose or oligosaccharide or inulin or lactulose of lactitol).mp.
29	23 or 24 or 25 or 26 or 27 or 28
30	exp Fecal Microbiota Transplantation/
31	(((feces or fecal or faecal or faeces or stool or microbio* or microflora) adj3 (transplant* or transfuse* or transfer* or therap* or treat* or implant* or instillation or donor*)) or FMT).mp.
32	((bacteria or bacterio*) adj2 (transplant* or transfuse* or transfer* or therap* or treat* or implant* or instillation or donor*)).mp.
33	30 or 31 or 32
34	exp Gastrointestinal Microbiome/
35	exp Microbiota/
36	((feces or fecal or faecal or faeces or gut or intestinal or gastrointestinal) and (microbio* or microflora))
37	34 or 35 or 36
38	exp Synbiotics/
39	synbiotic*.mp.
40	38 or 39
41	22 or 29 or 33 or 37 or 40
42	3 and 41
43	42 and 13

Key: *mp,* title, abstract, original title, name of substance word, subject heading word, floating sub-heading word, keyword heading word, organism supplementary concept word, protocol supplementary concept word, rare disease supplementary concept word, unique identifier, synonyms; *sh* MeSH subject heading, *ti* title, *ab* abstract, *pt* publication type, *fs* floating subheading

We will also search the online trial registries ClinicalTrial.gov (clinicaltrials.gov/) for potential information from unpublished and ongoing studies. Finally, the reference lists of all included studies will be reviewed to identify other relevant trials.

### Data collection and analysis

#### Selection of studies

**Fig. 1:**
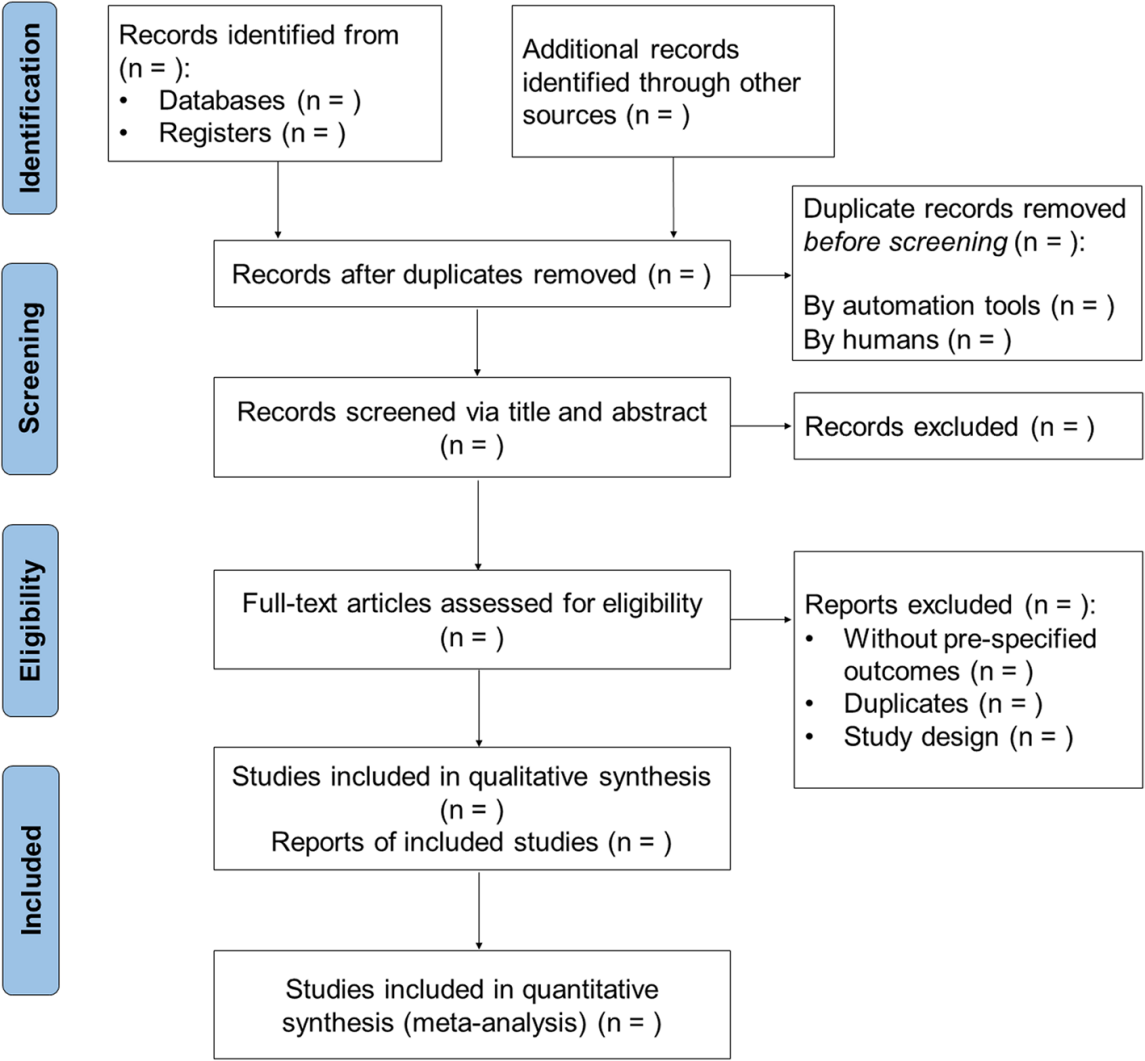
The PRISMA flow diagram of study identification and selection. PRISMA, Preferred Reporting Items for Systematic Reviews and Meta-Analyses

Two review authors (Honglin Jiang and Yan Peng) will independently perform the whole procedure of study identification and selection by Endnote X9 software (Clarivate Analytics, Boston, MA). Any disagreements will be resolved through consensus or consultation with a third author (Yibiao Zhou). Firstly, the authors will screen the results of electronic searches to identify the duplicate records that are not removed by automation tool. The titles and abstracts of non-duplicate reports will then be assessed for potential eligibility, and those obviously irrelevant or inappropriate studies will be excluded. Afterwards, the full texts of each potentially eligible trial will be retrieved and thoroughly reviewed for inclusion by both authors. For studies without a full-text content of link in the electronic databases, we will contact the corresponding author for a full-text copy via the attached email address. If trials are described in more than one report that all meet the inclusion criteria, we will link these reports together before data collection. The procedure of study identification and selection will be reported in a PRISMA flow diagram (Fig. 1).

#### Data extraction and management

Two independent authors (Honglin Jiang and Yan Peng) will extract data from each selected trial using a predesigned, standardized data extraction sheet, and created in Microsoft Excel 2019. The consistency of collected data will be checked and determined by a third reviewer (Yibiao Zhou). We will extract data including information as follows:

1. Trial characteristics: study design (cross-over or parallel), blinding, arm, first author, publication year, country of origin, inclusion criteria, study population, sample size, diagnostic criteria of cirrhosis, analyzing methods (intention-to-treat or per-protocol), and trial duration
2. Participants: mean age, gender distribution, etiology of cirrhosis, and complications
3. Intervention details: type of MTT, agent, does, duration, and mode of administration
4. Outcomes: as previously defined

#### Dealing with missing data

We will contact the original authors of selected articles to obtain any required information and clarify unclear data. Studies with missing data that cannot be acquired will be critically appraised for inclusion.

#### Assessment of risk of bias

Two authors (Honglin Jiang and Yan Peng) will assess the methodologic quality of included studies separately using the Cochrane Collaboration’s tool and classify the risk of bias as high, low, or unclear [20]. Inconsistencies will be discussed with a third author (Yibiao Zhou). The domains of the risk evaluation include the selection bias (random sequence generation and allocation concealment), performance bias (blinding of participants and personnel), detection bias (blinding of outcome assessment), attrition bias (incomplete outcome data), and reporting bias (selective reporting).

#### Data synthesis

We plan to undertake the meta-analysis using RevMan 5.4 software (Nordic Cochran Centre, Copenhagen, Denmark) and R 4.0.4 software (The R Foundation for Statistical Computing, Vienna, Austria). Clinical heterogeneity was assessed by grouping studies by study population characteristics (e.g., adults, cirrhosis stage), interventions, and outcomes. Studies will be quantitatively synthesized when there are ≥ 3 RCT reports within a single grouping. We prefer to compare all outcomes using the intention-to-treat principle. Cochrane *Q* test and *I*^*2*^ statistics will be used to assess the heterogeneity at study level. Data will be pooled using a random-effects model when *I*^*2*^ > 50% or *P* < 0.1; otherwise, a fixed-effects model will be applied to combined the results. The results of meta-analyses will be presented as risk ratios (RR) for categorial outcomes (e.g., rate of HE/MHE occurrence) and mean differences (MD) for continuous data (e.g., ALT, AST, and ALB) with 95% confidence intervals (CI). For some studies that only report the outcomes at the end of intervention or at endpoint, we will use the final time point available. If data are too heterogeneous to pool or not provided in a format suitable for pooling (e.g., data reported in different units of measurement that cannot be converted), we will use a narrative synthesis.

#### Assessment of heterogeneity

According to the *I*^*2*^ statistics value, the inter-study heterogeneity will be defined as unimportant (0–40%), moderate (30–60%), substantial (50–90%), and considerable (75–100%).

#### Subgroup analysis

Possible sources of heterogeneity will be explored through subgroup analysis and meta-regression when necessary. We plan to perform the pre-specified subgroup analyses based on the following, if feasible: type of MTTs and study population, primary outcomes, different versions of MELD, analyzing methods, and mean age (<18 versus ≥18 years). The intervention effects may be further investigated within each subgroup between different agent, does and duration of treatment if there are enough data.

#### Sensitivity analysis

We will perform sensitivity analysis to test the stability of each outcome result by removing several studies that may have a potential influence on the effect size. Trails will be excluded in a sensitivity analysis if (1) with high risk of bias in methodologic quality, (2) with considerable heterogeneity (*I*^*2*^ ≥75%), and (3) with insufficient data or have other features that recognized by at least two reviewers.

#### Assessment of publication bias

We will perform funnel plots and Egger regression asymmetry test when there are at least ten studies with the same outcome left to assess for the potential existence of publication bias and other small study effects.

#### Quality of the evidence

We will present a summary table containing the main outcomes of the review and their evidence grading, assessed by two authors (Honglin Jiang and Yan Peng) using Grades of Recommendation, Assessment, Development and Evaluation (GRADE) approach [21].

## Discussion

To the best of our knowledge, this review is the first to comprehensively assess the liver-specific effects of multiple MTT therapies in cirrhosis. The close interaction between the gut and the liver can be a major factor in the pathogenesis of liver damage and liver cirrhosis progression [2]. Many studies are being performed to suppress further liver fibrosis by modulating the gut microbiome. However, cirrhosis with different aetiologies varies in compositions of gut microbiota and mechanisms of developing liver fibrosis. The therapeutic effects of MTTs are inconsistent and need to be deeply studied in regard to their possible backgrounds. To ascertain the potential of gut-based therapy for treating cirrhosis, we plan to investigate the effect of common MTTs on liver function and disease severity. Our findings will provide more conclusive and stronger evidence about the efficacy of each gut microbiome-related intervention (probiotics, prebiotics, synbiotics, and FMT) on improving patients’ conditions via hints from liver indicator changes and also evaluate the role of MTTs in cirrhosis treatment from a whole insight. A potential limitation could be that studies focusing on antibiotics will not be included for analysis. This may impact the final evaluation of MTT effects. While probiotics, prebiotics, synbiotics, and FMT are commonly utilized to repopulate helpful bacteria in the host, antibiotics are used to fight bacterial infections and can result in indiscriminately killing symbiotic microbes. This side effect and the emergence of antibiotic resistance has long been appreciated. Thus, it may not be appropriate to combine the effect of antibiotics with other type of MTTs.

## Supplementary Information

**Additional file 1.** PRISMA-P 2015 Checklist. This checklist is used to check the procedure when preparing this protocol. It has been adapted for use with protocol submissions to Systematic Reviews from Table 3 in Moher D et al: Preferred reporting items for systematic review and meta-analysis protocols (PRISMA-P) 2015 statement. Systematic Reviews 2015 4:1.

## Data Availability

The datasets used and/or analyzed will be available from the corresponding author on reasonable request.

## Abbreviations

ALB: Albumin
ALT: Alanine aminotransferase
AST: Aspartate aminotransferase
BILI: Bilirubin
CENTRAL: Cochrane Central Register of Controlled Trials
CI: Confidence intervals
CTP: Child-Turcotte-Pugh
FMT: Fecal microbiota transplantation
GRADE: Grades of Recommendation, Assessment, Development and Evaluation
HE: Hepatic encephalopathy
IL: Interleukin
MD: Mean differences
MELD: Model for end-stage liver disease
MHE: Minimal hepatic encephalopathy
MTTs: Microbiome-targeted therapies
NAFLD: Non-alcoholic fatty liver disease
PRISMA-P: Preferred Reporting Items for Systematic Reviews and Meta-Analyses Protocols
RCT: Randomized controlled trials
RR: Risk ratios
SAE: Serious adverse events
SOC: Standard of care
TNF: Tumour necrosis factor
WBC: White blood cell counts
